# Smartwatch Detection of Refractory Hypoxemia in Phenazopyridine-Induced Methemoglobinemia: A Case Report

**DOI:** 10.1007/s13181-025-01090-9

**Published:** 2025-08-04

**Authors:** Leann N. Tulisiak, Mackenzie E. Loesing, Alexander Ljungberg, Eric E. Kaczor

**Affiliations:** Department of Emergency Medicine, Jacobs School of Medicine and Biomedical Sciences, University at Buffalo, State University of New York, Buffalo, NY USA

**Keywords:** Methemoglobinemia, Pulse Oximetry, Wearable Electronic Devices, Biosensors, Phenazopyridine

## Abstract

**Introduction:**

Significant cases of methemoglobinemia will cause a signal interference in traditional dual-wavelength pulse oximetry leading to the classic finding of “refractory hypoxemia”. As smartwatches begin to incorporate pulse oximetry capabilities, it is unclear whether these devices will demonstrate the same type of interference in the presence of methemoglobinemia. This article reports a case where a patient’s Apple Watch© reported a hypoxemia alert in the setting of phenazopyridine induce methemoglobinemia.

**Case Report:**

This is a case report of a 71-year-old female whose Apple Watch© reported a hypoxemia alarm in the setting of methemoglobinemia caused by phenazopyridine use. The patient’s smartwatch began signaling hypoxemia alerts the day before she noticed clinical cyanosis. 24 hours after the hypoxemia alerts began, she presented to the Emergency Department (ED) with shortness of breath and blue discoloration of skin. The Apple Watch© recorded oxygen saturation readings between 73% and 86%. Upon arrival to the ED, the patient’s SpO_2_ was 84% on traditional hospital-grade dual-wavelength pulse oximetry and her methemoglobin level was 19.8%. Her symptoms and methemoglobinemia resolved after administration of 1 mg/kg of IV methylene blue.

**Conclusion:**

Pulse oximetry used in smartwatch technology can trigger a hypoxemia alarm in the presence of drug-induced methemoglobinemia. If demonstrated to be a reproducible finding, smartwatch technology has the potential to be used as part of an early detection system for clinically significant methemoglobinemia.

## Introduction

Traditional dual-wavelength pulse oximetry calculates SpO_2_ by measuring the absorbance of specific wavelengths of light and using brand specific algorithms to distinguish between deoxyhemoglobin and oxyhemoglobin [1]. When these devices are used on patients with methemoglobinemia, they are prone to a computational error that results in reporting the presence of hypoxemia [1]. Methemoglobinemia is a condition that occurs when the heme group of the hemoglobin molecule is oxidized from a ferrous (Fe^2+^) to a ferric (Fe^3+^) state [2]. Several common medications have been implicated as potential causes of methemoglobinemia and include local anesthetics, nitrites, dapsone, and phenazopyridine [3–6]. Patients who develop significant methemoglobinemia usually present with symptoms of cyanosis and dyspnea and with hypoxemia on pulse oximetry that is refractory to oxygen supplementation due to this computational error. This phenomenon has been well-established for pulse oximeters used in the hospital setting, such that clinicians are trained to recognize it as a clue to help make the diagnosis of methemoglobinemia. There is emerging evidence that devices that incorporate pulse oximetry for everyday use, such as smartwatches, also exhibit this same interference and have helped prompt patients to seek medical care [4, 5]. This case report details an episode of phenazopyridine-induced methemoglobinemia where hypoxemia was noted by the patient’s Apple Watch© prior to presentation to the Emergency Department (ED).

### Case Report

A 71-year-old female with a past medical history of hypertension, myelofibrosis, bipolar disorder, depression, and recurrent urinary tract infections (UTIs) presented to the ED for dyspnea and decreased exercise tolerance. The patient reported normal exercise tolerance at baseline and frequently walked laps in a local mall for exercise. The day before presentation, she was walking in the mall when she was notified by her Series 6 Apple Watch© (version 9.6.3) that her oxygen level was low. She reported that she ignored the hypoxemia alert but began to experience mild dyspnea on exertion and had to stop more frequently than usual to catch her breath when walking. During the 24-hours from the initial hypoxemia alert to her ED presentation, the patient’s Apple Watch© reported oxygen saturations between 73% and 86%. On the morning of presentation, the patient’s family noted a blue discoloration of her skin which prompted her to seek care. On further investigation, the patient reported one week of dysuria and urinary urgency, for which she reported taking over-the-counter Azo (phenazopyridine hydrochloride). She initially took 100 mg three times per day for a total of seven days, however in the 24-hours prior presentation she took a total of 1200 mg for symptom relief– 800 mg over an 18-hour period the day prior to presentation and an additional 400 mg the morning of presentation.

Upon arrival to the ED, her vitals were SpO_2_ 84% on room air using traditional dual-wavelength pulse oximetry, heart rate 92 bpm, blood pressure 157/72mmHg, and temperature 36.7 C. She was not in respiratory distress but was placed on 15 L non-rebreather with minimal improvement in her oxygen saturation to an SpO_2_ of 89%. Her physical exam was notable for cyanosis with blue-tinged skin and lips but was otherwise unremarkable. The patient was found to have a methemoglobin level of 19.8% on venous blood testing. Her laboratory evaluation showed a white blood cell count of 12.2 × 10^9^/L, thrombocytosis, a hemoglobin of 10.4 g/dl which was around her baseline, a creatinine of 1.28 mg/dL, and a venous blood gas (VBG) demonstrated a pH 7.50, pCO_2_ 31mmHg, pO_2_ 30mmHg, and bicarbonate 25 mmol/L. Cardiac markers, coagulation studies, and chest x-ray were unremarkable. Her urinalysis was consistent with a UTI and she was given intravenous Ceftriaxone.

After discussion with the Poison Control Center, she was given 1 mg/kg of methylene blue for suspected methemoglobinemia secondary to phenazopyridine toxicity. Despite treatment, her SpO_2_ reading remained 88% while in the ED and she was admitted to the hospital. Approximately 6-hours after the administration of methylene blue she was asymptomatic and saturating in the mid-90s on room air. The Apple Watch© pulse oximeter readings during the hospitalization were not recorded. A second venous blood methemoglobin level was sent 7-hours after methylene blue administration and was 3.0%. A repeat VBG showed resolution of the respiratory alkalosis with a pH of 7.43, pCO_2_ 43mmHg, pO_2_ 58mmHg, and bicarbonate 22 mmol/L. She was discharged home approximately 24-hours after her initial presentation with oral antibiotics and proper dosage recommendations for phenazopyridine to prevent repeat methemoglobinemia. Consent for publication of this case was obtained and provided to the journal in accordance with *JMT* policy.

## Discussion

This case demonstrates that a Series 6 Apple Watch© (version 9.6.3) triggered a hypoxemia alert in a patient with phenazopyridine-induced methemoglobinemia. A similar instance of hypoxemia alerts before apparent cyanosis in the setting of anemia was also observed in another case report (methemoglobin level 11.4% and hemoglobin 9 g/dL) [4]. It takes a serum concentration of about 1.5 g/dL of methemoglobin to cause clinical cyanosis, which is around 10–20% methemoglobinemia depending on a person’s baseline hemoglobin concentration. Due to this range, some patients can get significant methemoglobinemia without developing the classic cyanosis that will prompt medical attention. Taken together, these case reports suggest that the Apple Watch© with pulse oximetry technology may be able to detect this significant methemoglobinemia without cyanosis. This phenomenon could be utilized to alert patients who have been exposed to a xenobiotic that induces methemoglobinemia and encourage them to seek care before their symptoms worsen and perhaps prevent further exposure.

While the two earlier case reports provide useful clinical background regarding other smartwatch hypoxemia alert events, those reports do not disclose the brand of smart watch or consider why these devices would trigger a hypoxemia alarm in the presence of methemoglobinemia [4, 5]. Traditional dual-wavelength pulse oximetry devices shine light through a patient’s skin (usually a finger) and measure the absorbance of light at wavelengths of 660 nm and 940 nm. Deoxyhemoglobin absorbs light better at a wavelength of 660 nm (red light) and oxyhemoglobin absorbs light better at a wavelength of 940 nm (near-infrared light). Using brand specific algorithms, these devices will determine the ratio of Red: Near-Infrared light that is absorbed to distinguish the amount of deoxyhemoglobin and oxyhemoglobin in the patient’s blood [1]. Microprocessors in medical grade pulse oximeters then use this ratio to calculate SpO_2_ over a series of pulses such that a higher ratio (Red: Near-Infrared light absorption) correlates with a lower SpO_2_ (because deoxyhemoglobin absorbs more red light) and vice versa [1, 7]. These algorithms are not designed to consider the presence of methemoglobin. Methemoglobin absorbs both near-infrared and red light equally well. As such, in the presence of significant methemoglobinemia the ratio of Red: Near-Infrared absorbance approaches 1, which correlates to an SpO_2_ of approximately 80–85% [1, 7]. The minimum percentage or concentration (g/dl) of methemoglobinemia necessary to achieve this ratio for each brand of device is unclear. Another method used to detect methemoglobinemia is co-oximetry. Masimo© is a common brand of co-oximetry used in the hospital setting, and the user manual for their Radical-7© Pulse CO-Oximeter© reports that it utilizes “7 +” wavelengths of light from “various visible and infrared lights with LEDs 500nm to 1400nm” [8, 9]. Since the specific wavelengths of light used for this technology are proprietary, it is difficult to make a meaningful comparison between the technologies.

The Apple Watch© Series 6 in this case uses red, infrared, and green LEDs for its various health features, operating at wavelengths of approximately 660 nm, 850 nm, and 525 nm, respectively [10]. The red and infrared wavelengths are used for pulse oximetry and have been shown to be reliable in comparison with medical grade oximeters [11]. Interestingly, while the difference between the infrared wavelengths in the traditional pulse oximetry and the Apple Watch© was not slight (940 nm vs. 850 nm), the methemoglobin interference appears to still be present in the Apple Watch© technology. Other smartwatches have also begun to include pulse oximetry features as well, including the Garmin Venu 3©, FitBit Sense 2©, Samsung Galaxy Watch 6©, Polar Vantage V3©, and G-Shock Move©. While the Garmin© watches use red and infrared wavelengths at 630–660 nm and 940 nm, respectively [12], the wavelengths of red and infrared light used by these other brands are currently proprietary information and not available for comparison. Given this 90 nm difference in infrared wavelengths used between traditional pulse oximetry and the Apple Watch© technology, the unknown brands of smartwatches demonstrating interference from methemoglobinemia in the two other case reports cited, and that all these smartwatch devices rely on a similar measuring technique, it is quite possible that these other smartwatch brands will also be susceptible to this phenomenon of methemoglobin interference. If the generalizability of this phenomenon is proven to be true, smartwatches may have the potential to become a ubiquitous tool that could monitor for clinically significant methemoglobinemia, and perhaps sulfhemoglobinemia as well given the similar mechanism of interference outlined above [1]. This would be especially useful if such a monitoring tool could somehow be paired with a patient’s medication list and factor in when they are prescribed medications known to put them at risk for these conditions.

It is unclear why the patient in our case had persistent hypoxemia on hospital pulse oximetry in the ED. While methylene blue is known to cause changes in the SpO2 readings [13], this effect is likely short lived. A retrospective chart review of 103 patients who received 100 mg of methylene blue as part of their care during thoracoabdominal aortic repair was performed with the goal of characterizing the duration of time it took for the SpO2 to drop and recover after methylene blue administration [13]. They found that SpO2 recovered to baseline at about 6 min after administration. This timeline is not consistent with the prolonged pulse oximetry readings in our case. Interestingly, the authors of this paper speculate that “Any substance that absorbs significantly at the wavelengths of 660 or 940 nm may interfere with the accuracy of the pulse oximeter reading.”. Given this, it is likely that methylene blue would also cause an interference in smartwatch pulse oximetry according to the mechanism we have laid out.

### Limitations

This case report has several limitations. First, this single case prevents a full characterization of the described interference in smartwatch pulse oximetry, including the minimum amount of methemoglobinemia necessary to cause it. Second, the details of symptom onset and medication dosing are potentially limited by recall bias, and we do not have serial methemoglobin levels with corresponding hospital pulse oximetry and Apple Watch© pulse oximetry readings. Most smartwatch brands and the common brand of co-oximeters keep the wavelengths of light that they use for their devices proprietary, making direct comparison difficult. While Apple does publicly report the approximate LED wavelengths used in its technology, the authors of this work do not have access to the proprietary algorithms for the technology leading to uncertainty on how exactly it works. 

## Conclusions

Our case adds to the growing body of case report literature demonstrating that the pulse oximetry used in smartwatch technology can trigger a hypoxemia alarm in the presence of drug induced methemoglobinemia. More work is needed to understand the pulse oximeter technologies being deployed in smartwatches and explore the limits and reproducibility of their ability to detect methemoglobinemia. If demonstrated to be a reproducible finding, smartwatches have the potential to help detect xenobiotic induced methemoglobinemia and prompt victims to seek medical attention.

## Declarations

### Previous Presentations

Data in this manuscript were previously presented at ACMT’s Annual Scientific Meeting, Washington, DC, 2024.
